# Control of Partial Coalescence of Self-Assembled Metal Nano-Particles across Lyotropic Liquid Crystals Templates towards Long Range Meso-Porous Metal Frameworks Design

**DOI:** 10.3390/nano5041766

**Published:** 2015-10-26

**Authors:** Ludovic F. Dumée, Jean-Baptiste Lemoine, Alice Ancel, Nishar Hameed, Li He, Lingxue Kong

**Affiliations:** Deakin University, Geelong, Australia, Institute for Frontier Materials, 75 Pigdons Road, Waurn Ponds Vic 3216, Australia; E-Mails: jblemoin@deakin.edu.au (J.-B.L.); aancel@deakin.edu.au (A.A.); nishar.hameed@deakin.edu.au (N.H.); li.he@deakin.edu.au (L.H.); lingxue.kong@deakin.edu.au (L.K.)

**Keywords:** meso-porous metal framework, lyotropic liquid crystals templating, metal nano-particle coalescence, SAXS ordered phase shifts, interface nano-particle coalescence

## Abstract

The formation of purely metallic meso-porous metal thin films by partial interface coalescence of self-assembled metal nano-particles across aqueous solutions of Pluronics triblock lyotropic liquid crystals is demonstrated for the first time. Small angle X-ray scattering was used to study the influence of the thin film composition and processing conditions on the ordered structures. The structural characteristics of the meso-structures formed demonstrated to primarily rely on the lyotropic liquid crystal properties while the nature of the metal nano-particles used as well as the their diameters were found to affect the ordered structure formation. The impact of the annealing temperature on the nano-particle coalescence and efficiency at removing the templating lyotropic liquid crystals was also analysed. It is demonstrated that the lyotropic liquid crystal is rendered slightly less thermally stable, upon mixing with metal nano-particles and that low annealing temperatures are sufficient to form purely metallic frameworks with average pore size distributions smaller than 500 nm and porosity around 45% with potential application in sensing, catalysis, nanoscale heat exchange, and molecular separation.

## 1. Introduction

Meso-porous metal frameworks, with average pore size distributions below 500 nm, exhibit unique surface to volume ratios whereby the metal natural’s optical and catalytic properties and ductility become dramatically enhanced [1]. Typically, through sufficient increase of the relative density of metal nano-particles (NPs) assembled across an otherwise porous matrix, non-linear optical responses are an order of magnitude higher than that predicted by effective medium theories [2,3]. Similarly, partially coalesced metal NP frameworks offer electrical conductivity higher by up to 10-fold compared to the bulk metal while the thermal conductivity increased by three-fold compared to non-coalesced nano-particle networks [4,5]. While recent work demonstrated the formation of meso-porous metal structures by selective de-alloying of thin film alloys [6] and cold-compression of mixed silica-metal powders [7], metal meso-porous foams, and electroless deposited structures are currently the most commonly applied materials for development of novel electrodes, heat sink, or separation materials, because they are generally more easily to scale up [8]. By far, the most notable progress has been achieved through a deeper understanding of coalescence mechanisms and the development of advanced routes to stabilize metal nano-particles into ordered and porous ordered micro-networks [1,9]. Further improvement to the stability of long range ordered metal nano-particle assemblies and enhancement of pore density and homogeneity are, however, still pressing issues [10,11].

Lyotropic liquid crystals (LLC) self-assembled metal NP frameworks represent one newly developed methods to stabilize nano-porous metal frameworks. The dispersion of NPs across a semi-rigid sacrificial ordered template, typically an amphiphilic polymer gel [12,13,14,15,16,17,18,19] is a highly efficient route to process meso or nano-scale materials. This technique offers much greater crystallinity and pore morphology control than conventional nano-fabrication techniques, such as foaming or de-alloying, due to the natural ordered properties of the templating LLC matrix. Importantly, such self-assembled metal NP frameworks can be directly deposited onto other porous materials, including ceramics or polymers, thereby providing excellent nano-/micro-scale interfaces and cohesion [20,21]. Recently, efforts to increase the long range stability and decrease the average pore size distribution of nanoporous metal and metal oxide [22] frameworks have led to highly promising architectures for fast heat dissipation [23], energy generation [24], sensing [25], or molecular fractionation [1,26,27,28,29]. Nevertheless, such nano-porous structures that integrate the properties of the bulk metal on long range order and maintain those obtained at the nanoscale have not yet been achieved. To this end, it is essential to fabricate ultra-thin materials that are highly accessible through pores, activated surface area, and enhanced phonon propagation properties usable as storage and separation materials, or as active interfaces for energy transfer and lab-on-a-chip devices.

Self-assembly is a spontaneous process whereby molecules organize via non-covalent interactions between macro-molecular polymeric chains [3,30]. LLCs may arrange into various ordered or semi-ordered long range order structures, including spherical, lamellar, cubic, or hexagonal structures due to variations of process parameters, such as concentration in solution, type of solvent, average molecular weight of the different LLC phases, weight ratio of the different blocks composing the LLC, as well as solution pH or temperature [3,18]. LLCs have been used as sacrificial templates for the integration of silica [31] and carbon nanotubes [32]. Although the integration of the carbon nanotubes was shown to induce a change in the ordered structure of the LLC, this method offers interesting insights using potentially cheap and biodegradable LLCs. While ligand-stabilized platinum NP assemblies were successfully self-assembled with *in situ* synthesized poly (isopreneblock-dimethylaminoethyl methacrylate) or PI-b-PDMAEMA [12], the fabrication of purely metallic porous frameworks from such a route has yet to be demonstrated.

Here, commercially available Pluronics LLCs were used for the first time as sacrificial templates to form meso-porous metal structures by incorporation of metal NPs into the hydrophilic domain of the ordered LLC matrix and controlled thermal coalescence at low temperature. The incorporation in the LLC ordered phase was shown to alter the ordered structure of the LLC and lead, under annealing controlled conditions, to a purely metallic meso-porous material. The impact of the annealing conditions on the stability of the ordered phases formed upon NPs incorporation across the LLC matrix will be discussed in light of the final material morphology and small angle X-ray scattering (SAXS) data and for the first time related to the dynamic of single particles coalescence.

## 2. Results and Discussion

**Figure 1:**
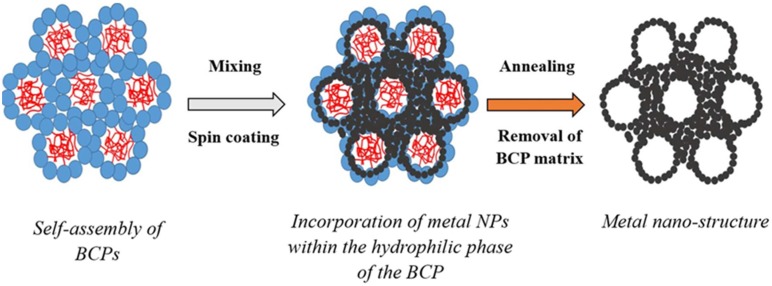
Schematic of the formation of the metal nano-porous networks by templating metal nano-particles (NPs) across self-assembled block-copolymer (BCP) materials assembled as lyotropic liquid crystals (LLC) without pore collapse.

The self-assembly of LLC micelles in solution leads to the formation of long range ordered phases [2]. The nature and homogeneity of the phases as well as the ordered parameters of the different ordered phases may be evaluated by SAXS analysis [33,34]. SAXS patterns analysis offers invaluable insights to both the homogeneity of the crystallinity of a network and to potentially locate order-disorder transitions. Ordered phases can therefore be identified by analysing the ratios of the different scattering vector *q_i_* over the value of the first scattering vector q* corresponding to their Bravais Lattice parameters [32]. The simultaneous removal of the LLC system and coalescence of hydrophilic NPs should lead to a meso-porous system where pores are formed within the sites of the initial hydrophobic particles (Figure 1).

**Figure 2:**
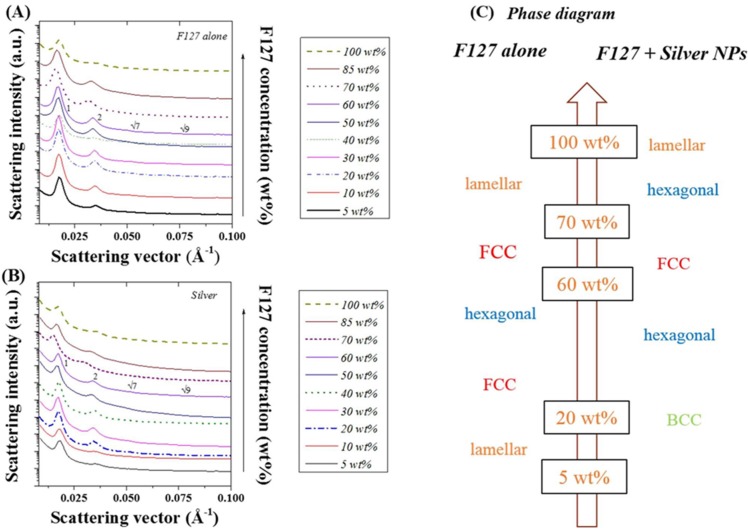
Small angle x ray scattering (SAXS) spectra obtained for (**A**) F127 in water at different LLC/water ratios; (**B**) after addition of 20 nm diameter silver NPs (15 wt %); and (**C**) corresponding ordered phases obtained from peak ratio identification (Table S1).

Lattice systems, such as lamellar, hexagonal, or body-centred cubic, exhibit a specific scattering signature relying on the long range order of the main geometrical features (Figure 1). Examples of the values of the ratios *q_i_*/q* for the main ordered phases encountered for LLC are presented in Table S1. Figure 2 correlates the SAXS scattering patterns obtained to the LLC (Pluronic F127) ordered structure for a series of LLC/water weight ratios. As previously reported ([32,35,36] and Figure 2A), the ordered phases of F127 mixed in water undertake a number of shifts depending on the relative concentration of LLC to water. Here, the ordered phase changes from lamellar at concentrations below 20 wt%, to FCC (20 and 40 wt %), hexagonal (40 and 60 wt %), prior to re-shifting to face centred cubic (FCC) (60 to 70 wt %) and lamellar (70 to 100 wt %) (Figure 2C). As seen in Figure 2B, the incorporation of 20 nm silver NPs (20 wt %) strongly shifts the ordered structure. Furthermore, the ordered phase shift from BCC below 20 wt %, to hexagonal (20 to 60 wt %), FCC (60 to 70 wt %), hexagonal (70 to 85 wt %), and lamellar (above 85 wt %) (Figure 2C). Peaks scattering vectors and intensities for the series of samples are presented in Table S2 and Table S3 for the F127 and Ag NP F127 systems respectively. The integration of spherical metal NPs into the LLC/water system did have a strong impact on the structure formed as previously demonstrated for tubular CNT additives in [32]. Although not all the peaks referenced in Table S1 are present for all the samples, the number of distinct peaks, at least three to four, are representative of a specific ordered structures. Based on Figure 2A, three peaks may be distinguished at F-127/water ratios of 30, 50 and 85 wt %. When determining the *q*/q* ratios, these three concentrations observe peak ratios at 1, √4, √9, and √15. At 40 and 70 wt % F-127/water, peak ratios observed are 1:√4:√7:√9 representative of a hexagonal structure. Interestingly, these results, at the edge of two different ordered phase regions, slightly differ to those previously reported in [32] which may be attributed to slight water losses. These variations, potentially localized, may therefore lead to concentration variations across the samples as the film deposition techniques used in [32] differs from our spin coating technique.

**Figure 3:**
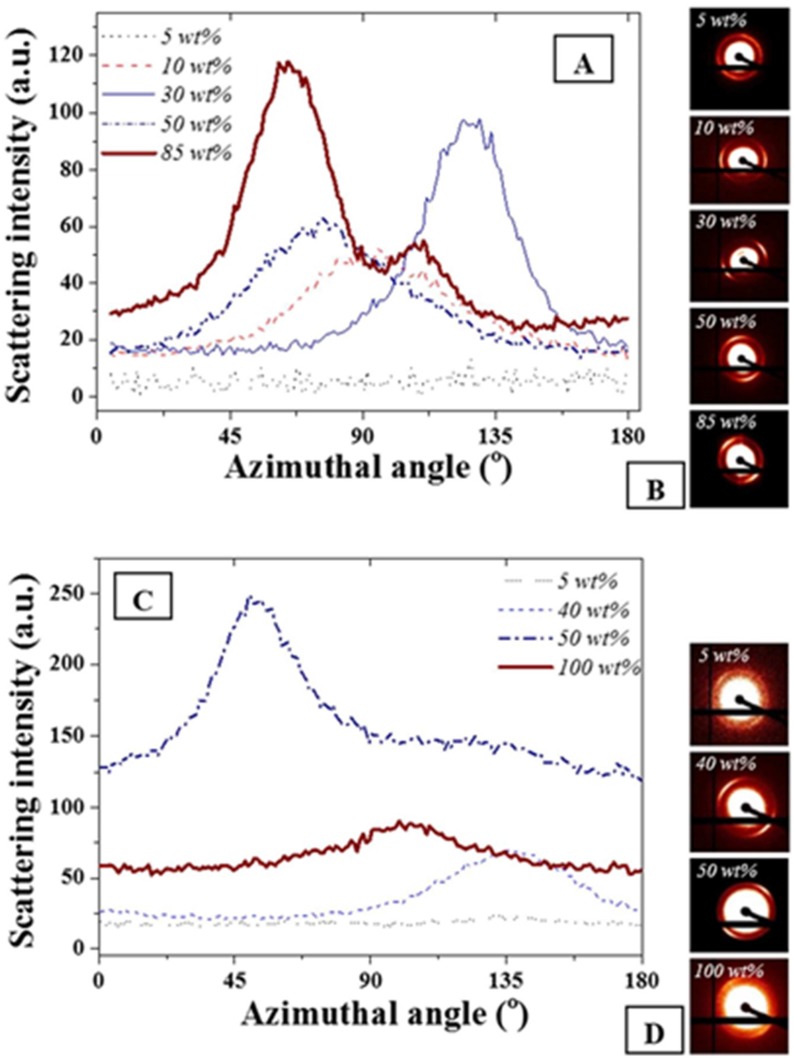
(**A**) Azimuthal angle variations for F127 in water at different F127 in water concentrations; (**B**) corresponding scattering images visually demonstrating asymmetry of the distribution; (**C**) F127 with 15 wt % NPs in water at similar wt% and (**D**) corresponding scattering images. The weight ratios not represented here all exhibited similar trends as the 5 wt % where no azimuthal angle variation was detected for the peak at 0.015 Å^−1^. The azimuthal angles were calculated following a procedure detailed in [37].

The azimuthal angle was evaluated for the main scattering peak at 0.015 Å^−1^. As expected and seen in Figure 3, no variations of full width at half maximum were detectable on the main diffraction peak for the FCC and BCC ordered phases due to geometrical symmetries. The presence of the anisotropies [34,37] was clearly visible on the scattering images shown in Figure 3B,D. However, for both F127 in water and F127 in water with NPs, slight variations of the cylindrical phases formed by the hexagonal structure or of the layered structures formed for the lamellar structures were found. The strongest anisotropy were found at 85 and 50 wt % F127 in water and the F127 silver NP systems respectively. Interestingly, the 85% F127 in water also presents a clear pattern asymmetry. This could be related to the presence of multiple phases, including potentially a mixture of hexagonal and lamellar phases. The formation of these phases could be due to the spin coating process or to localized grain misorientations due to relatively poor mixing of the NPs across the LLC template. Micron-sized grains are typically found for such LLC mixtures rendering the long range stabilization of the structure challenging [12,13].

**Figure 4:**
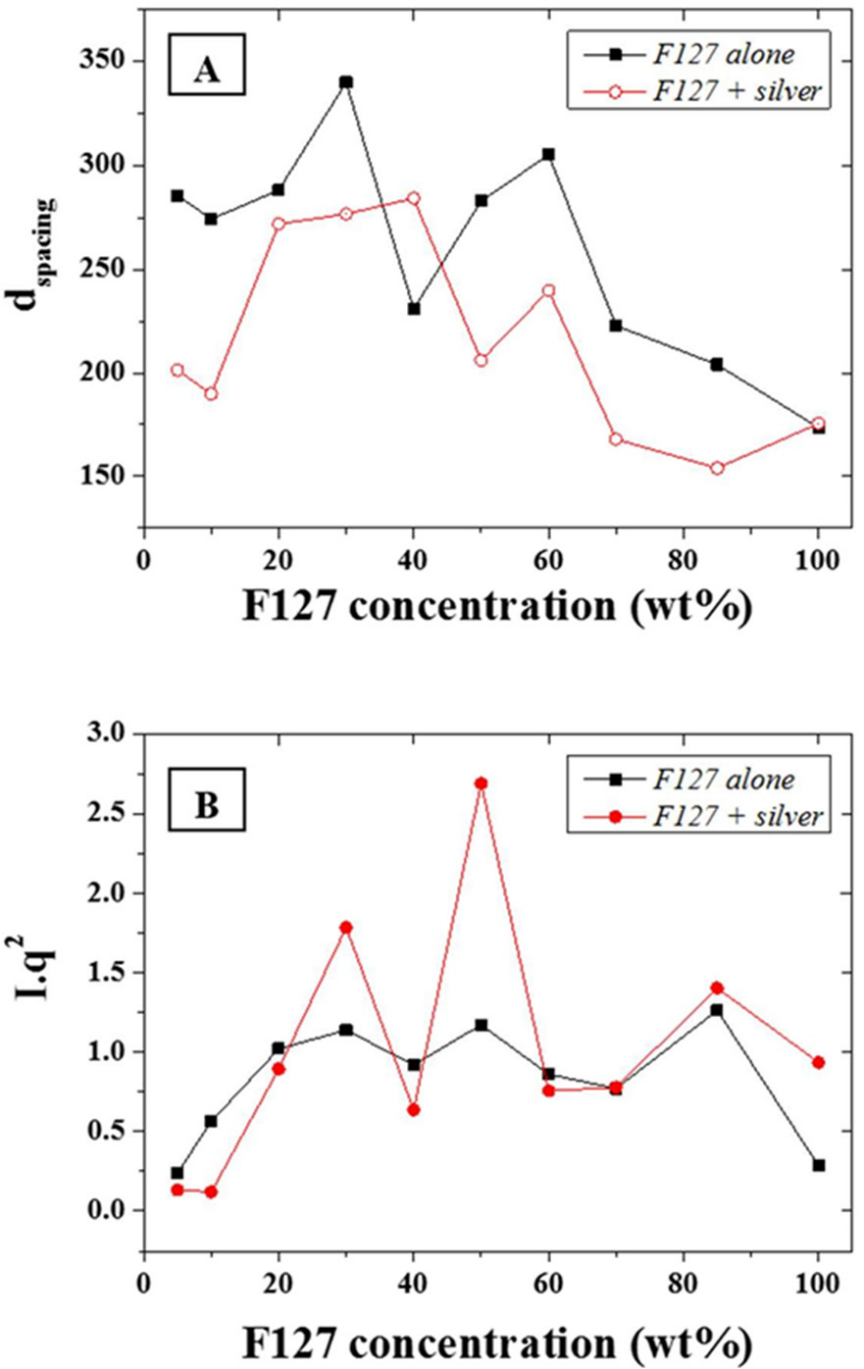
(**A**) d-spacing in Angstrom (Å) for the F127 series with and without addition of silver NPs; and (**B**) Calculated I*q^2^ for the same series.

The d-spacing, *d*, of the main scattering peak, calculated from the approximation *d*~2*π*/*q*, (0.015 Å^−1^—Figure 4A) was shown to be constant at ~30 nm for F-127 in-water concentrations between 10 and 50 wt % while it sharply decreased above 50 wt % down to 20 nm. The d-spacing, or inter atomic plane distance, provides information about the diffraction of large nanostructures, as given by Bragg’s law, stating that at certain specific wavelengths and incident angles, crystals produced intense peaks of reflected radiation known as Bragg peaks and at small angles (below 5°) [33]. The presence of the silver NPs led to a slightly narrower d-spacing distribution with a very similar trend to the series without NPs. This suggests that that the concentration of F127 in water does not affect the size of the micelles forming in solution from 10 to 50 wt % of LLC in water and that the aqueous spacing across the hydrophilic domains of the micelles are therefore sufficiently large to accommodate the metal NPs without dramatically compromising the ordered structure but through slightly compressing the meso-structure of the micelles. The distance between the micelles, which composes most of the hydrophilic domains for the F127, is directly related to the LLC local concentration and therefore more likely to be affected by the presence of the NPs than the micelle size. The latter is only dependent on the surfactant morphology and the LLC packing density. Hence, micelles formed with F-127 in water will form meso-structures of roughly the same size independently of the LLC concentration while their morphology may change. In order to resolve potential morphological shifts, the I*q^2^ of the main scattering peaks across the range 0.015 to 0.1 Å^−1^ of the F-127 alone in water and with NPs were evaluated (Figure 4B). The calculation of I*q^2^, provides information of structural factors weighted by the average number of scatterers in the distribution. Furthermore, although the scattering intensity was expected to rise continuously with the relative LLC concentration increase in water, this was tempered by localized thickness variations of the samples cast on silicon wafers. This was attributed to the high viscosity of the LLC gels at high LLC loadings rendering spin-coating difficult to perform. The variations of I*q^2^ as function of the F-127/water loading with silver NPs was found to follow a similar trend to the system without silver NPs. Three main peaks may be observed respectively at ratios of 30, 50, and 85 wt %. The analysis of the peak ratios obtained at LLC concentrations of 30, 50, and 85 wt % indicate an FCC structure is obtained at 50 wt % (peak ratios of 1:2:√8:√16) while hexagonal structures are found at 30 and 85 wt % (peak ratios of 1:2:3 and 1:2:√7 respectively). These results show that it is possible to obtain a nano-scale hexagonal structure when incorporating metal nanoparticles of 20 nm diameter into a LL/water system. Thermo gravimetric analysis (TGA) was performed in N_2_ to determine the impact of the Ag NP integration in the LLC matrix on the decomposition temperature of F-127. The degradation temperature of F-127 was found to vary between 350 and 400 °C depending on the loading of NPs (Figure S1). The presence of silver NPs was shown to slightly reduce the thermal stability and shift the start of the decomposition temperature by 20 °C at 30 wt %. In order to visually assess the nature of the ordered structures, trials to acquire Transmission Electron Micrographs (TEMs) were performed. However, no satisfactory images could be obtained due to necessary water content across the LLC system to maintain the ordered structure. Water was being instantaneously evaporated in the TEM due to the high vacuum necessary to stabilize the electron beam. The phases, clearly visible on the SAXS patterns could therefore not be imaged by TEM for this non cross-linked LLC NP system.

Furthermore, as seen in Figure S2, the nature of the metal NPs added to the LLC/water system does not evidently impact the meso-structure. The ordered phase for the samples prepared from similar sized silver, nickel, and copper NPs remained unchanged at the same volume %. However, variations of the d-spacing of the 1st peak of the SAXS patterns were found, and larger d-spacing were found for smaller particles, suggesting an impact of the particle diameter on the ordered pattern dimension. This result suggests that only the size of the metal NPs may impact the structure in the matrix. The larger the metal NPs are, the more impact it will have in the hydrophilic region of the LLC as their integration in the matrix will disturb the volume ratio between the hydrophilic and hydrophobic groups across the LLC and hence change the final material structure.

**Figure 5:**
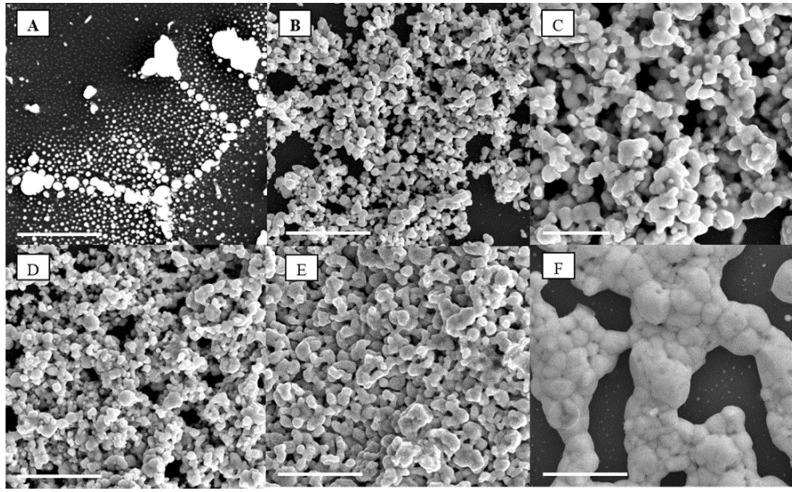
Scanning electron microscope (SEMs) of the silver NPs percolation threshold for (**A**) 1 wt %, (**B**) 4 wt %, (**C**) 10 wt %, (**D**) 20 wt %, (**E**) 30 wt %, and (**F**) 50 wt % after annealing in air at 250 °C; the scale bar on all images corresponds to 5 µm.

As shown in Figure 5 and Figure S3, the percolation threshold was attained for NP concentration higher than 5 wt % leading to a semi-continuous porous network, after annealing at 250 °C. The structure is shown to slightly densify above this percolation threshold as more silver NPs are integrated and coalesce during annealing. Annealing of the samples was performed at different temperatures to assess the impact on the ordered structure and metal NP coalescence mechanisms. The amount of NPs present in the structure was also checked by TGA, which showed reasonable agreement with theoretical calculations of sample concentrations (±5%) based on mixing ratios (Figure S1). Variations may, however, be attributed to the spin-coating process whereby NPs may be ejected by centrifugal force from the sample, locally altering the NP concentration. The duration of the annealing plateau was however not found to cause significant morphological changes above 1 h of annealing at 250 °C (Figure S4) suggesting that upon initial coalescence the system quickly reaches a steady state of minimum energy. This was also supported by *in situ* TEM thermal coalescence tests, performed in vacuum, where agglomerates were found to start forming between 100 and 250 °C (Figure S5). This trend was well supported by the SAXS modelling of the Guinier knees presented in Figure S6 and of the average scatterers dimension variation for an *in situ* thermal treatment.

**Figure 6:**
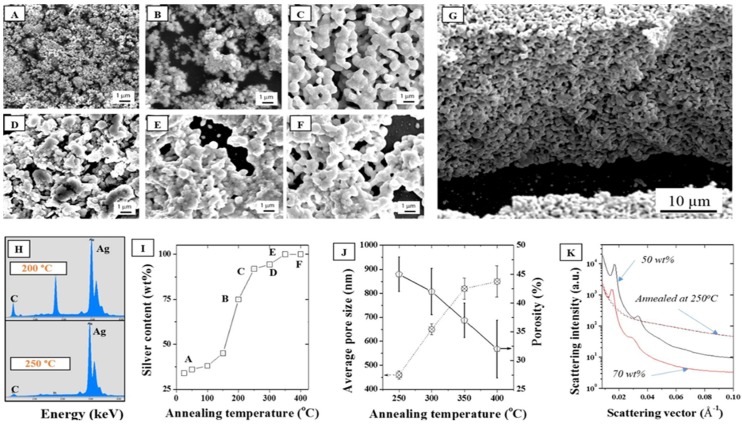
SEMs of a 30 wt % F127 sample in water with 15 wt % silver NPs after annealing in air at (**A**) 25, (**B**) 200, (**C**) 250, (**D**) 300, (**E**) 350, and (**F**) 400 °C, (**G**) Cross section corresponding to (**C**), (**H**) EDS elemental distributions at 200 °C and 250 °C showing a strong reduction of the carbon peak above 250 °C; (**I**) Corresponding silver surface content (EDS); (**J**) Average pore size and porosity determined by SEM analysis following for the series of samples (**A**–**F**); (**K**) Demonstration of the impact of annealing on the collapse of the hexagonal structure visible for two samples (50 and 70 wt %) before and after annealing at 250 °C.

In addition, the impact of the annealing temperature in air on the stability of the ordered phased upon LLC removal was performed to correlate the porosity formation to the coalescence mechanisms. The packing density and thus the nature of the interface between the NPs was therefore shown to be critical to the formation of homogeneous and narrow distribution pores across the surface and thickness of the material. As seen from the SEMs in Figure 6A–F, all the LLC was removed above 250 °C, at least 100 °C lower than determined with TGA, (Figure 6C) suggesting a dominant role of O_2_ on the LLC oxidative degradation. This was confirmed by Energy Dispersive Spectroscopy (EDS) where the elemental distribution of C/Ag was shown to drop dramatically above 250 °C to less than 1 wt % (Figure 6H,I). Furthermore, annealing tests performed in N_2_ (Figure S7) were shown to lead to much less removal at the same temperature than oxidations in air. Well ordered, fully metallic pores were formed above that temperature across the whole thickness of the samples (Figure 6G). Direct porosity and specific surface area measurements were carried out on the samples by helium pyknometry and BET N_2_ adsorption. However, the low volume fraction and high density of the samples, due to the metal presence and the ultra-thin structure of the samples deposited on the silicon wafers, did not allow for direct measurements and these techniques, typically applied to organic materials considered as inappropriate in this case. The average pore size distribution was therefore extrapolated from the SEMs and shown to increase by ~93% between 250 and 400 °C, while porosity decreased from 45% to 32% over the same temperature range. This collapse of the pore size distribution clearly underlines the particle coalescence mechanisms happening at low sintering temperatures. Pores at 250 °C appear to be evenly distributed across the thickness of the micron thick samples (Figure 6G). Annealing at lower temperatures than 250 °C was also performed in order to minimize structural change and limit coalescence but issues with remaining carbon materials remained, rendering the thin films highly fragile.

Furthermore, as seen in Figure 6K, the annealing process completely removed any trace of the hexagonal meso-structure visible on the non-annealed samples. Non-annealed samples show at least four distinct diffraction peaks and Guinier knees within the 0.01–0.06 Å^−1^ range while the carbonized samples only show a broad knee spread around 0.05 Å^−1^. It is hence clearly visible that the removal of the LLC has an impact on the structure. No peak is visible after carbonizing the samples at 250 °C which is likely due to a clustering of the silver NPs via thermal sintering when carbonizing the samples and collapse of the initial particle distribution. The sintering of the NPs is irremediable due to the high surface energy of the metal [38] and to the large specific surface area of the NPs [39]. The surface energy, *ε*, of the NP surface (Equation (1)) is primarily dependent on the ordered microstructure of the metal and on its energy of sublimation.

(1) $$\epsilon =\frac{\Delta Hs}{0.5N_{A}Z}$$

where ∆*Hs* is the molar enthalpy of sublimation, *N_A_* the number of atoms in one mole crystal, and *Z* the coordination number.

The maximum surface energies calculated by the Full Charge Density model (FCD) show that silver has a lower surface energy than copper or nickel, suggesting a lower tendency for coalescence than the other more reactive metals. Although the structure and crystallinity of the surface grains on the metal NPs may vary, the metals belonging to the p-block of the periodic table appear to be more inclined to sinter at lower temperature (Figure S8). This indicates that although no major differences of the LLC templated thin films were visible between the samples made of different NPs, annealing should lead to very different samples morphologies and therefore properties. The sintering process of metal nano-particles is in fact dominated by both Ostwald ripening and coalescence, two distinct and competing mechanisms [40]. While Ostwald ripening happens upon the exchange of individual atoms between growing and shrinking particles in direct contact, coalescence results from the relative motion and merge of adjacent NPs into one single entity. Although differentiating the two mechanisms here is difficult, the analysis of the SEMs in Figure 6 seems to indicate that the even nodule size distribution across the sintered material should be the result of coalescence only. The size of these nodules shows that an approximate 5 to 20 particles agglomerated to form well defined nodules. The contact and interface between the particles, largely due to the overcoming percolation threshold and relative contact surface across the LLC matrix should therefore lead the interfacial coalescence mechanisms. It is therefore expected that to reach smaller pore size distributions, a trade-off between LLC/NP concentration should therefore be reached to maintain the LLC ordered structure while allowing for sufficient NP to NP contact to allow for fine surface coalescence.

**Figure 7:**
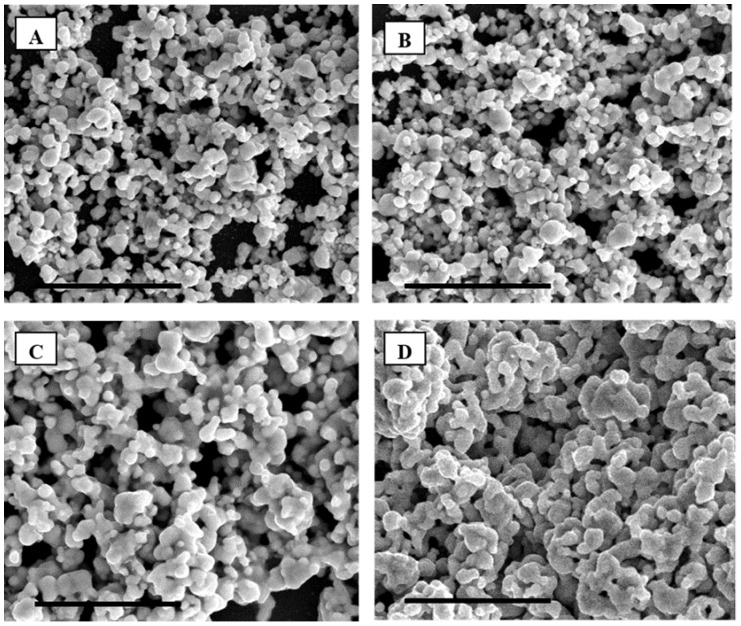
SEM of F127 in water with 15 wt % silver NPs spin-coated at respectively (**A**) 0, (**B**) 100, (**C**) 1000 and (**D**) 2000 rounds per minute (rpm) after annealing in air at 250 °C. The scale bar corresponds to 5 µm.

As seen on the SEMs presented in Figure 7, it appears that at spin-coating speeds of 1000 and 2000 rounds per minute (rpm), the silver NPs are ejected to the side of the silicon wafer (Figure S9), where NP clusters are formed after annealing at 250 °C. This phenomenon is not visible at lower spin-coating speeds, 100 rpm, where the film created appears much more homogeneous. Samples prepared without spin coating by direct casting of the gels were found to be very brittle and to exhibit cracks in the film. In addition, the average grain size of the metal clusters was found to increase with increasing spin-coating speeds (Figure 7). This phenomenon might be related to a better mixing and de-agglomeration of the NPs in the gel during spin coating. The centrifugal force acting as an effective way to prevent particle agglomeration and homogeneously disperse them across the LLC matrix. A rotating speed of 100 rpm therefore appeared as the most adequate to obtain a homogeneous film over the silicon wafer with very even distributions across large surface areas.

## 3. Experimental Section

### 3.1. LLC—Metal NP Sample Preparation

The LLCs were purchased from Sigma Aldrich and were used as received. The LLC used was a Pluronics F127. The metal NPs were purchased from Nanoamor (TX-USA) and used as received. Three NPs were used in this work including silver (99.9% pure, 20 nm, with approximately 0.3% PVP coating), copper (99.8% pure, 25 nm, passivated), and nickel (99.9% pure, 20 nm, carbon coated). A three-step process was followed in order to create nano-porous metal frameworks. The dispersion of the lyotropic liquid crystal with the nanoparticles in a suitable solvent; followed by casting of the resulting solution or gel onto a silicon wafer with subsequent carbonization of the thin film to remove the lyotropic liquid crystal.

The dispersion is essential for the formation of micelles in water. Direct mixing of LLC, nanoparticles and water was done at ambient temperature and neutral pH so as to simplify the system. Vigorous stirring for 1 to 3 h ensured a good homogeneity of the solution. For concentrations of LLC in water above 15%, a gel directly forms and it is impossible to stir correctly. Hence to reach concentrations as high as 70% LLC in water to obtain the hexagonal nanostructure, an evaporative method was employed. All samples were then prepared at 15% LLC/water and stirred as previously mentioned. Water was then evaporated to raise the concentration of LLC and hence obtain the desired hexagonal structure. Different LLC/water ratios were investigated for three different LLCs.

Dip and spin coating of the sample solutions on 5 × 5 mm silicon wafers were then performed. For spin-coating, the initial procedure was done at a rotational speed of 1000 rpm for 1 min on a single wafer spin processor (Laurell WS-400E6NPP, Laurell, PA, USA). Tests at different rotational speeds were done as well to verify the impact on NP stability and resulting metal frameworks. The carbonization step was performed in a tube furnace in air at 1 atm. The impact of the carbonization temperature on the structure was also investigated.

### 3.2. Characterization Techniques

SEM analysis and *in situ* cross sections were acquired and milled on a FEI Quanta 3D FEG electron—gallium dual beam (FEI Company, Hillsboro, OR, USA). The samples were carbon-coated prior to SEM analysis to obtain a better image quality. Images were taken at 5 keV and 10 mm working distance. EDS was performed at 20 keV, 10 mm working distance and with an EDAX detector on samples without carbon coating. Milling with the Ga FIB was performed at 20 keV and in three steps, including a rough milling step at 14 nA and two cleaning steps at 1 nA and 0.3 nA. Image analysis, thickness of the films, and statistics were performed with the software GIMP version 2.8. An average BET surface area was determined by N_2_ adsorption on a Micromeritics Tristar 3000 (Micromeritics Instrument Corporation, Norcross, GA, USA). The samples were first degassed for 70 h at 120 °C and then analysed at 77 K. TGA analysis was performed on a NIETZCHE-STA 409 PC (Nietzsche Company, Germany). Samples were spin-coated on a silicon wafer and evaporated for 3 h at 50 °C so as to remove the water present in the system. Spin-coated samples were then introduced in ceramic pans prior to analysis. The heating ramp was fixed at 10 °C/min from 100 to 450 °C. Transmission Electron Micrographs (TEMs) were captured on a Jeol 2100 LaB6 instrument (JEOL, Tokyo, Japan). The beam current was 110 µA and the accelerating voltage 200 keV. Samples are prepared by drop deposit on a copper grid. The TEM holder which was used was a heating stage (SmartSet Hot stage controller model 901, Gatan Incorporated, Pleasanton, CA, USA) which allowed to heat up the sample *in situ* within the chamber between room temperature and 500 °C. The *in situ* coalescence of silver NPs was studied by dispersing the particles either in ethanol or in the LLC phase. The solutions were dipped on TEM grids and left for drying for at least 1 h at room temperature prior to imaging. For the study in ethanol a solution of 0.5 wt % of NPs in ethanol was deposed on a copper grid. The sample was studied at different temperatures (25, 100, 200, 300, and 400 °C) and kept 25 °C before analysis. A solution of 1 wt % of LLC in water with a weight ratio of NP:LLC of 1:1 was analysed at different temperatures (25, 100, 200, 300, 400, and 500 °C). Each temperature was kept for 15 min before analysis. For both experiments the heating rate was of 50 °C/min and coalescence of the NPs were observed in function of the temperature. The SAXS beam-line at the Australian Synchrotron was used with a 1.6 m camera length to investigate the scattering patterns of different LLCs with and without NPs. The q range was from 0.008 to 0.5 Å^−1^. An In-vacuum undulator source (22 mm period, 3 m length maximum, Kmax 1.56) at a beam energy of 11 or 20 keV was used. Tests were performed at 25 °C unless otherwise specified.

## 4. Conclusions

In conclusion, the synthesis of meso-porous metal frameworks was demonstrated by controlled metal NP templating with common, commercially available LLCs. In addition, while the ordered structure of the self-assembled LLC was shown to be affected by the addition of the metal NPs, and slight compression of the hexagonal micelles were noticeable, the nature of the metal NPs did not seem to affect the morphology of the micelles. Although the ordered structure was not maintained after carbonization due to coalescence mechanisms between metal NPs, pore size distributions below 500 nm were homogeneously formed across micron thick films. The spin-coating speed was also shown to affect the final morphology of the samples and low spin coatings should be preferred to form smaller grains. The interface between the particles is found to be critical to finely control the coalescence process and mechanism towards smaller pore size materials design. Heat and mass transfers at the nano-scale should be investigated to further push the envelope of LLC template metal materials synthesis. These results are highly promising in order to fabricate nano- or mesoporous metal frameworks with potential application in separation, sensing, thermal exchanger or low temperature catalytic processes where nano-porous or nano-textured materials are preferred.

## Supplementary Materials

Supplementary materials can be accessed at: http://www.mdpi.com/2079-4991/5/4/1766/s1.
