# Supplementary material for: Control of Partial Coalescence of Self-Assembled Metal Nano-Particles across Lyotropic Liquid Crystals Templates towards Long Range Meso-Porous Metal Frameworks Design
Source: Nanomaterials (Basel). 2015 Oct 26;5(4):1766–81. doi: 10.3390/nano5041766 (PMC5304782; doi:10.3390/nano5041766)
Supplement: Supplementary file 1 [file nanomaterials-05-01766-s001.pdf]

Supporting Information

S1. SAXS Details and Theory

As seen in Equation (S1), the scattering vector (q, $Å^{-1}$) is determined from the beam wavelength characteristics (λ, cm^{-1}) and the scattering angle (θ, rad) formed from X-ray arrays scattered from features present across the sample:

$$q = \frac{4\pi}{\lambda}\sin(\frac{\theta}{2}) \quad (S1)$$

The d-spacing is defined in Equation (S2):

$$n\lambda = 2d\, sin\theta \quad (S2)$$

where n is an integer, λ is the wavelength of the incident beam, d is the spacing between the planes in the atomic lattice and θ is the angle between the incident ray and the scattering planes. At small angles (below 5°), the d-spacing may be evaluated as the inverse of the scattering vector q (Equation (S3)):

$$d = \frac{2\pi}{q} \quad (S3)$$

S2. Figures and Tables

Table S1. Examples of the values of the ratios q_i/q^* for the main crystalline phases encountered for block-copolymer (BCP); determination of the crystalline phases from the ratio q_i/q^* ratios obtained from small angle X ray scattering (SAXS) patterns analysis [26].

Structure	Face centred Cubic (FCC)	Body Centred Cubic (BCC)	lamellar	hexagonal
q/q*	√3: 2: √8: √11: √12: √16: √19	1: √2: √3: 2: √5: √6: √7	1: 2	1: √3: 2: √7: 3

Table S2. Structure parameters for F127 alone at different concentrations of BCP in water.

BCP concentration	Peak number	Scattering vector	Scattering intensity	√ q/q*	Structure analysis
wt%		Å^{-1}	a.u.	-	
**5**	q*	0.01782	750.66	1	lamellar
	2	0.03429	20.3099	3.702708907	
**10**	q*	0.017396	1862.22	1	lamellar
	2	0.03429	30.2394	3.885403904	
	3	0.0511889	9.67073	8.658702061	
**20**	q*	0.017395	3374.26	1	FCC
	2	0.0342925	57.8131	3.88641728	
	3	0.0501951	11.703	8.326716556	
	4	0.0690776	8.5966	15.76977926	
**30**	q*	0.0174	3760.96	1	FCC
	2	0.03429	43.5455	3.883617717	
	3	0.05218	10.4606	8.993104769	
	4	0.06808	8.30209	15.30878055	
**40**	q*	0.017395	3040.968	1	hexagonal
	2	0.0342925	15.8489	3.88641728	
	3	0.0541705	8.75206	9.697880364	
**50**	q*	0.017395	3873.95	1	FCC
	2	0.0332986	74.7347	3.664401758	
	3	0.0492012	15.0265	8.000230947	
	4	0.0660963	9.6786	14.43794661	
**60**	q*	0.01693	3006.16	1	hexagonal
	2	0.0332986	58.6658	3.868459307	
	3	0.0501951	12.2531	8.790401895	
	4	0.0660963	8.95361	15.24194469	
**70**	q*	0.015484	3215.5	1	hexagonal
	2	0.0313107	58.329	4.089019944	
	3	0.04224	16.4431	7.441859968	
	4	0.0501951	12.4329	10.50887732	
	5	0.0680838	8.77771	19.3339793	
**85**	q*	0.01649	4643.99	1	FCC
	2	0.032046	82.5937	3.776646581	
	3	0.0501951	17.017	9.265766172	
**100**	q*	0.017395	942.631	1	hexagonal
	2	0.0342925	48.6684	3.88641728	
	3	0.0511889	24.5871	8.659697629	

Table S3. Structure parameters for F127 and the Ag nano-particles (NPs) at different concentrations of BCP in water.

BCP concentration	Peak number	Scattering vector	Scattering intensity	√ q/q*	Structure analysis
wt%		Å^{-1}	a.u.	-	
5	q*	0.01797	410.849	1	BCC
	2	0.03529	19.0263	3.856622783	
	3	0.04423	11.5553	6.058117948	
10	q*	0.01781	378.728	1	hexagonal
	2	0.03429	20.0448	3.706868086	
	3	0.05318	9.55342	8.915974692	
20	q*	0.01736	2969.43	1	hexagonal
	2	0.03429	50.5903	3.901535191	
	3	0.0502	12.381	8.361958207	
	4	0.06808	8.78721	15.3794092	
30	q*	0.01713	6082.49	1	hexagonal
	2	0.03429	94.4827	4.007008321	
	3	0.05119	16.2395	8.930083776	
	4	0.06709	10.6874	15.33913458	
40	q*	0.01753	2071.66	1	FCC
	2	0.03429	46.3521	3.826230577	
	3	0.05119	11.4321	8.527199562	
	4	0.06808	8.62888	15.08256696	
50	q*	0.0167	9648.42	1	FCC
	2	0.0333	213.901	3.976083761	
	3	0.04721	41.2285	7.991624296	
	4	0.0661	17.5666	15.66642762	
60	q*	0.01716	2571.18	1	hexagonal
	2	0.03429	63.2894	3.993010049	
	3	0.05318	14.4249	9.604219984	
	4	0.06908	10.2278	16.20578567	
70	q*	0.014477	3713.45	1	lamellar
	2	0.02932	104.252	4.101765077	
	3	0.05616	14.4972	15.04863295	
85	q*	0.0164	5219.64	1	hexagonal
	2	0.033299	126.767	4.122534064	
	3	0.04324	39.0198	6.951582391	
100	q*	0.0174	3087.21	1	lamellar
	2	0.03529	102.783	4.113436716	
	3	0.05815	29.7025	11.16865669	

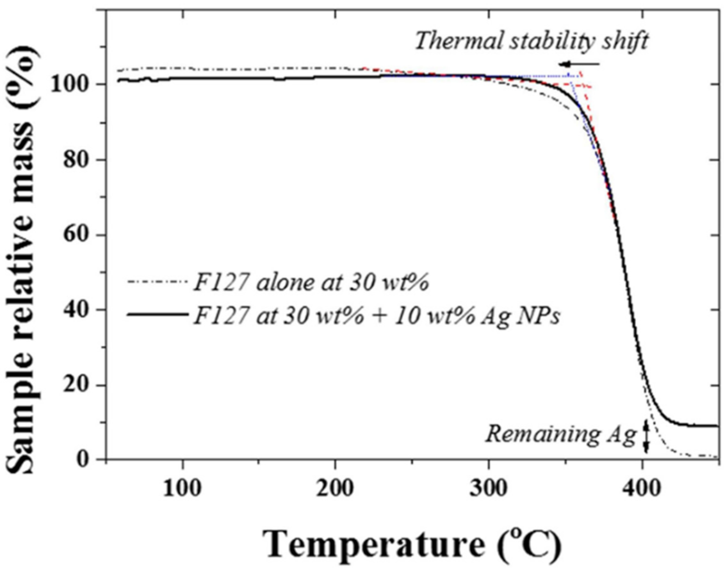

Figure S1. Thermo gravimetric analysis (TGA) of F127 and F127 with 30 wt % silver NPs.

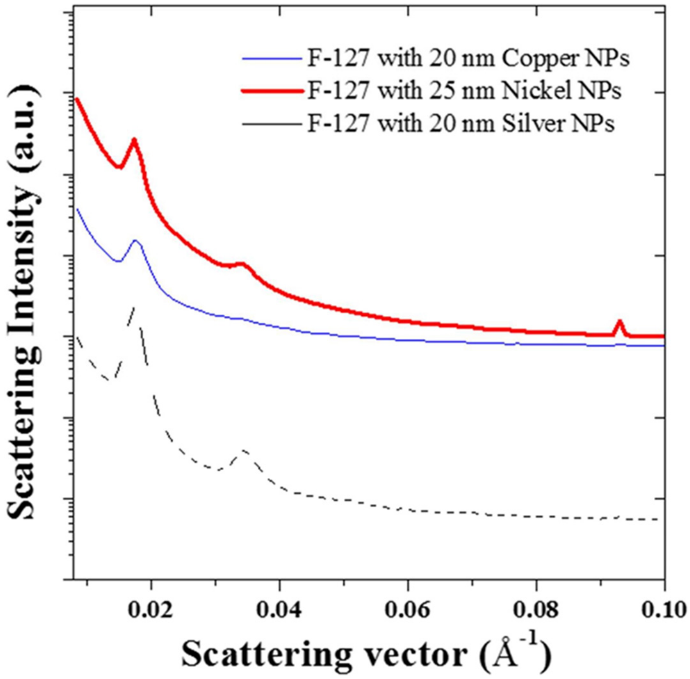

Figure S2. Impact of different metal NP (silver, copper and nickel) of similar size on the crystalline structure of the lyotropic liquid crystals (LLC) micelles.

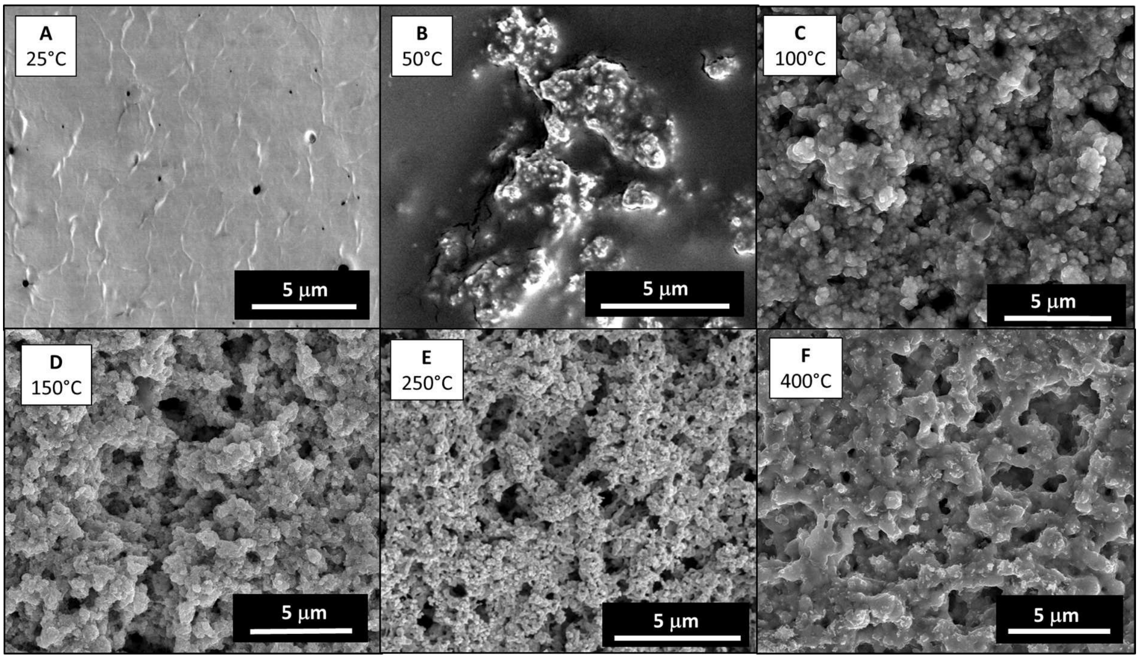

Figure S3. Impact of the temperature on the final morphology and removal of the LLC template. The temperatures used included (**A**) 25 °C, (**B**) 50 °C, (**C**) 100 °C, (**D**) 150 °C, (**E**) 250 °C and (**F**) 400 °C for fixed durations and conditions as described in the experimental section.

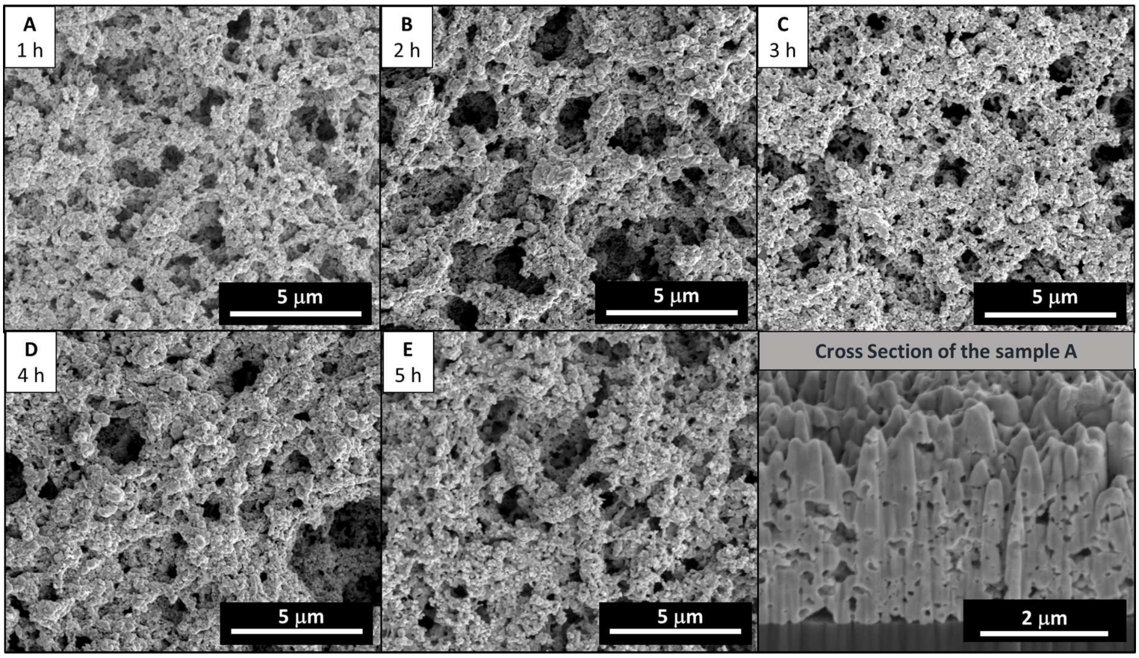

Figure S4. Impact of the annealing duration at 250 °C on the morphology of the structure for (**A**) 1 h, (**B**) 2 h, (**C**) 3 h, (**D**) 4 h and (**E**) 5 h. (**F**) is a representative cross section of (**A**). Tests performed for conditions presented in the experimental section.

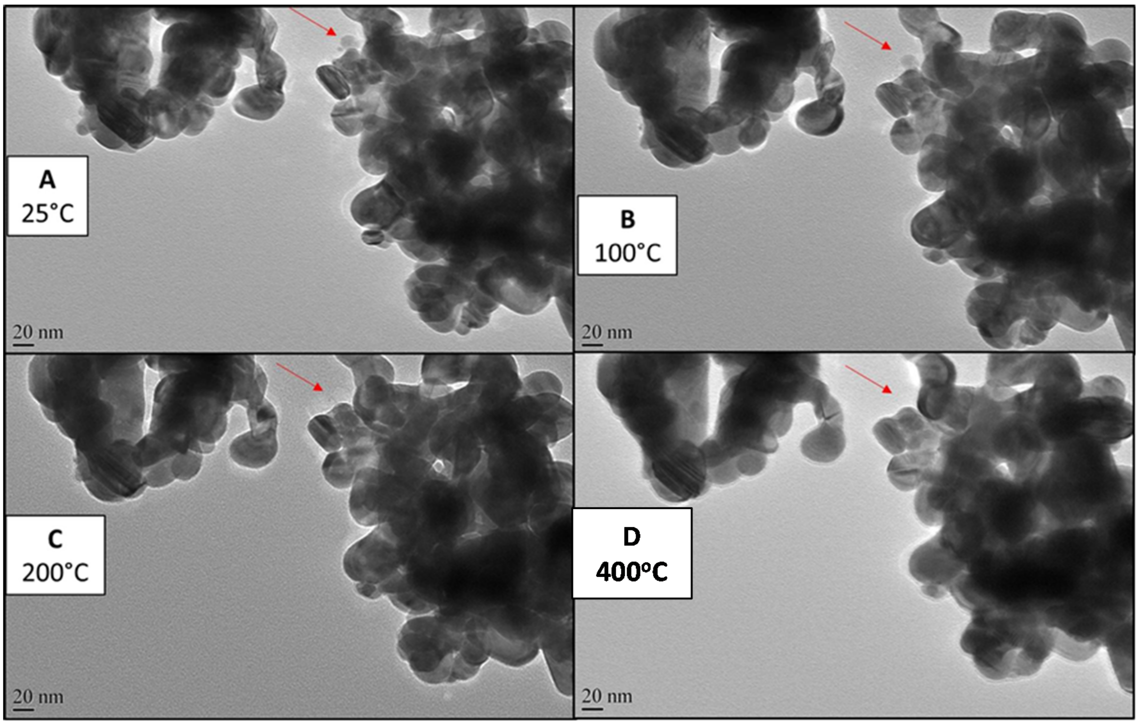

Figure S5. *In-situ* transmission electron micrograph (TEM) coalescence experiments performed with a for a 0.5 wt % of NPs in ethanol mixture at (**A**) 25 °C, (**B**) 100 °C, (**C**) 200 °C and (**D**) 400 °C.

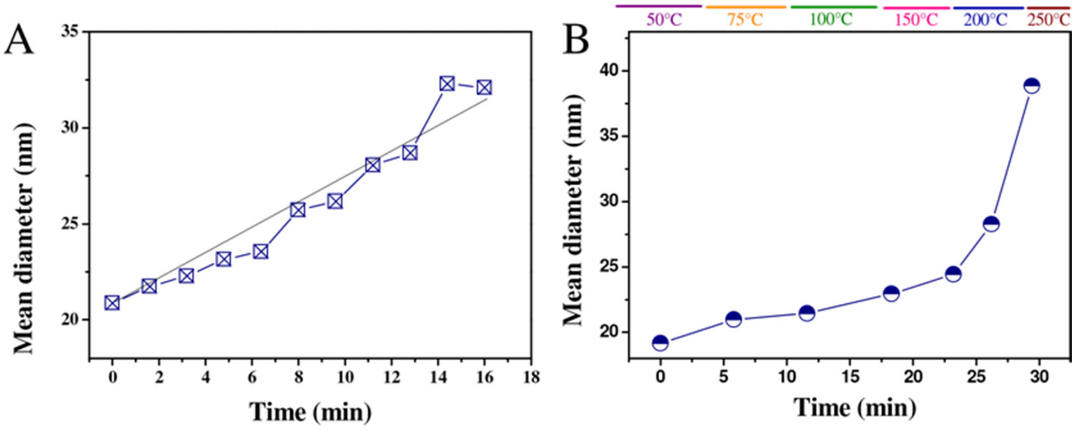

Figure S6. SAXS modelling for the average scatterer dimension as a function of (**A**) time of annealing in air at 50 °C; and (**B**) temperature of annealing over time.

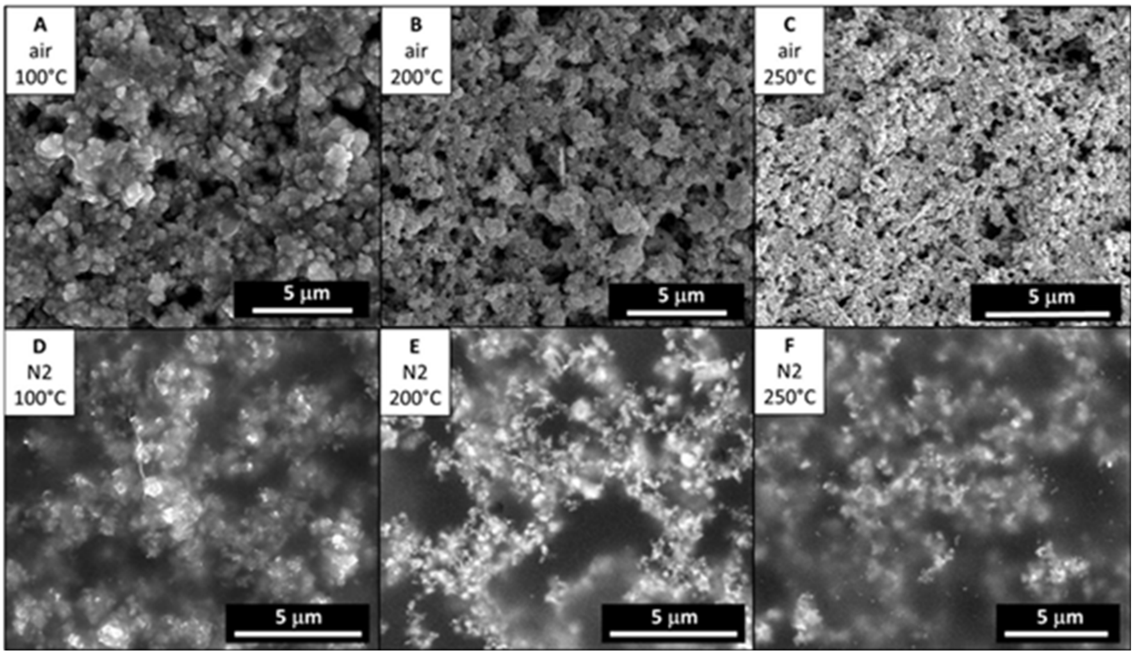

Figure S7. Impact of the annealing atmosphere on the LLC removal and pore formation in air and N_2.

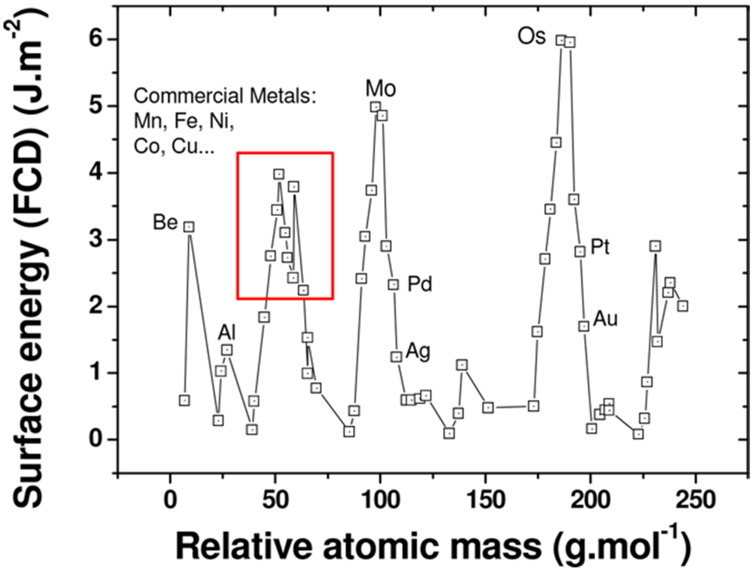

Figure S8. Surface energies of metal surfaces computed by full charge density model (FCD) as a function of the materials density [34].

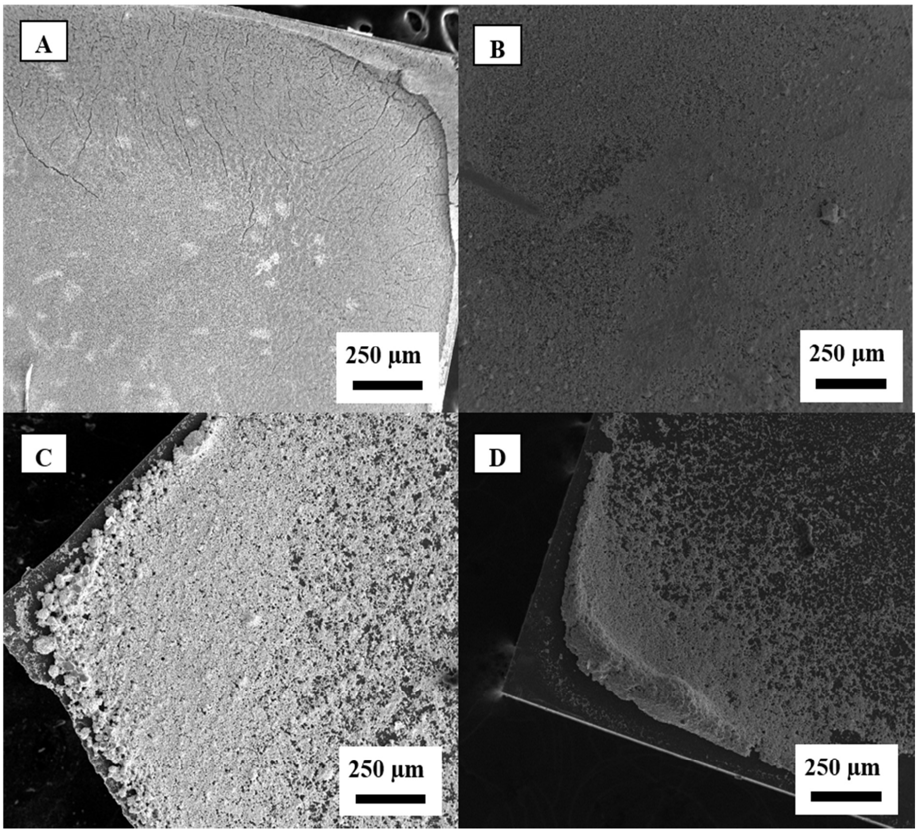

Figure S9. Evidence of particle ejection during spin coating for the same spin coating conditions presented in Figure 7 at (**A**) 0 rpm, (**B**) 100 rpm, (**C**) 1000 rpm, and (**D**) 2000 rpm.

© 2015 by the authors; licensee MDPI, Basel, Switzerland. This article is an open access article distributed under the terms and conditions of the Creative Commons Attribution license (http://creativecommons.org/licenses/by/4.0/).
